# TGF-β/Smad3 signalling regulates the transition of bone marrow-derived macrophages into myofibroblasts during tissue fibrosis

**DOI:** 10.18632/oncotarget.6604

**Published:** 2015-12-14

**Authors:** Shuang Wang, Xiao-Ming Meng, Yee-Yung Ng, Frank Y. Ma, Shuang Zhou, Yang Zhang, Chen Yang, Xiao-Ru Huang, Jun Xiao, Ying-Ying Wang, Shuk-Man Ka, Yong-Jiang Tang, Arthur C.K. Chung, Ka-Fai To, David J. Nikolic-Paterson, Hui-Yao Lan

**Affiliations:** ^1^ Li Ka Shing Institute of Health Sciences, Departments of Medicine and Therapeutics, Chemical Pathology, and Anatomical and Cellular Pathology, The Chinese University of Hong Kong, Hong Kong SAR, China; ^2^ Division of Nephrology, Department of Medicine, Institute of Clinical Medicine, Taipei Veterans General Hospital, National Yang Ming University, Taipei, Taiwan; ^3^ Department of Nephrology and Monash University Department of Medicine, Monash Medical Centre, Clayton, Victoria, Australia

**Keywords:** TGF-beta, Smad3, macrophage-myofibroblast transition (MMT), lineage tracing, renal fibrosis

## Abstract

Myofibroblasts are a main cell-type of collagen-producing cells during tissue fibrosis, but their origins remains controversial. While bone marrow-derived myofibroblasts in renal fibrosis has been reported, the cell origin and mechanisms regulating their transition into myofibroblasts remain undefined. In the present study, cell lineage tracing studies by adoptive transfer of GFP+ or dye-labelled macrophages identified that monocyte/macrophages from bone marrow can give rise to myofibroblasts via the process of macrophage-myofibroblast transition (MMT) in a mouse model of unilateral ureteric obstruction. The MMT cells were a major source of collagen-producing fibroblasts in the fibrosing kidney, accounting for more than 60% of α-SMA+ myofibroblasts. The MMT process occurred predominantly within M2-type macrophages and was regulated by TGF-β/Smad3 signalling as deletion of Smad3 in the bone marrow compartment of GFP+ chimeric mice prevented the M2 macrophage transition into the MMT cells and progressive renal fibrosis. *In vitro* studies in Smad3 null bone marrow macrophages also showed that Smad3 was required for TGF-β1-induced MMT and collagen production. In conclusion, we have demonstrated that bone marrow-derived fibroblasts originate from the monocyte/macrophage population via a process of MMT. This process contributes to progressive renal tissue fibrosis and is regulated by TGF-β/Smad3 signalling.

## INTRODUCTION

Fibrosis is a key pathological feature and is considered to be a final common pathway leading to end-stage organ failure in many chronic diseases regardless of the underlying aetiology [1]. The principal cell type responsible for increased deposition of fibrillar collagen during active tissue fibrosis are myofibroblasts – a subset of activated fibroblasts characterized by expression of alpha-smooth muscle actin (α-SMA) [2, 3]. Myofibroblasts are a heterogeneous population which may be derived from more than one precursor population, [4] including epithelial-mesenchymal transition (EMT) [5–8], endothelial-mesenchymal transition (EndoMT) [9–11], and local proliferation of resident fibroblasts or pericytes [12, 13].

An extra-renal origin for myofibroblasts has also been established through studies in chimeric mice showing that bone marrow-derived cells can differentiate into collagen I producing cells myofibroblasts during renal fibrosis [14–20], although not all studies have confirmed this finding [12, 21]. In addition, the chemokine CXCL16 and chemokine receptor CXCR6 are required for the recruitment of bone marrow-derived fibroblasts into the fibrosing kidney [22, 23]. However, a number of questions remain unresolved, including; (i) do bone marrow-derived fibroblasts come from the macrophage compartment, (ii) does the transition of bone marrow-derived macrophages to fibroblasts occurs within the fibrosing kidney, and (iii) what is the mechanism governing this transition?

This study used the well-characterised unilateral ureteric obstruction (UUO) model of renal fibrosis to address these questions. We present evidence based upon cell transfer studies that bone marrow macrophages can undergo a process of macrophage-myofibroblast transition (MMT) within the fibrotic kidney. Chimeric mice and *in vitro* studies defined a requirement for TGF-β/Smad3 signalling in the transition of bone marrow-derived macrophages into collagen-producing myofibroblasts. In addition, we suggest that MMT occurs more prominently in M2-type macrophages.

## RESULTS

### Chimeric studies identify bone marrow-derived myofibroblasts in renal fibrosis

**Figure 1:**
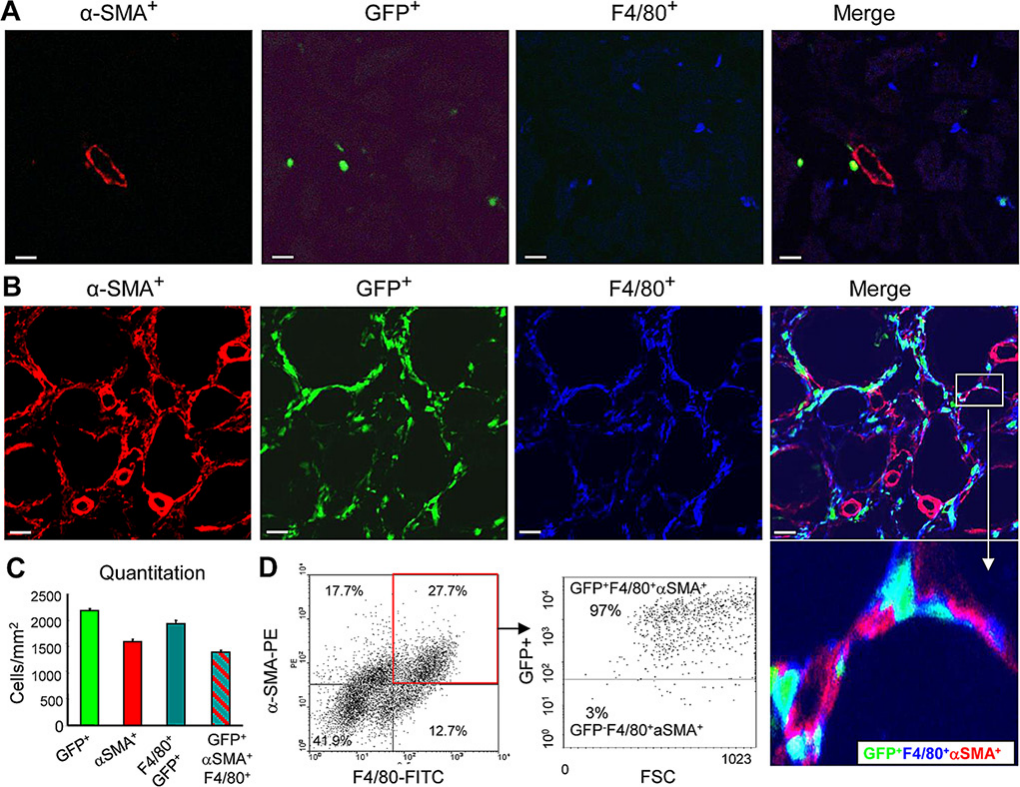
Bone marrow-derived myofibroblasts express macrophage markers in the UUO model (**A-C**) Lethally irradiated mice were reconstituted with GFP^+^ bone marrow cells, and 8 weeks later underwent a 7 day UUO. (A) Confocal microscopy of the contralateral control kidney shows α-SMA expression in an arteriole (red), the presence of GFP^+^ cells (green), and F4/80^+^ macrophages (blue). Note, most F4/80^+^ resident macrophages within the control right kidney lack GFP. (B) The obstructed kidney contains many interstitial α-SMA^+^ cells (red) that co-express GFP (green), and F4/80 (blue). An inset illustrating triple labelled GFP^+^α-SMA^+^F4/80^+^ cells are clearly shown. (C) Quantification of the cell populations in the UUO kidney based on confocal microscopy. (**D**) Three-color flow cytometric analysis of cells isolated from the UUO kidney showing that the majority of α-SMA^+^ myofibroblasts co-express the F4/80 macrophage antigen (^+^α-SMA^+^F4/80^+^ cells), and most of these cells also express GFP indicating a bone marrow origin. Data represent results from groups of 6 animals and each bar resents mean ± SEM. Scale bar, 20 μM.

**Figure 2:**
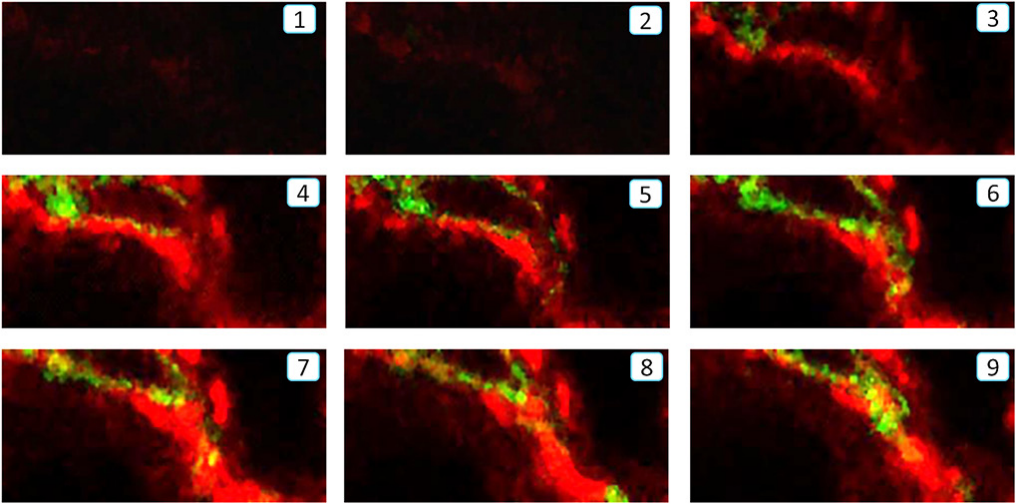
Z-stack analysis of a single F4/80+ α-SMA+ cell undergoing the MMT process in the fibrotic kidney of UUO by confocal microscope Z-stack analysis shows a sequence of 9 slices of the image in the Z-plane illustrating a MMT cell undergoing the MMT process by co-expressing F4/80 (green) and α-SMA (red) antigens in the UUO kidney, which is further illustrated by a video image in the Supplementary file.

To establish whether a subset of α-SMA^+^ myofibroblasts in the fibrosing kidney derive from the bone marrow compartment, we performed a chimera study in which C57BL6 mice were lethally irradiated and then injected with bone marrow cells from transgenic C57BL6 mice that constitutively express the green fluorescence protein (GFP). Following reconstitution for 8 weeks, chimeric mice underwent the UUO procedure. Compared to the contralateral control kidney, confocal microscopy identified a population of α-SMA^+^ myofibroblasts that co-expressed GFP and F4/80, indicating a process of MMT from bone marrow macrophages (Figure 1A–1C). This was confirmed by examination with Z-stack confocal microscope, showing a single F4/80+α-SMA+ cell under the process of MMT (Figure 2 and Supplementary video). Further studies by flow cytometric analysis of single cell suspensions prepared from enzyme-digested kidneys showed that a substantial population of α-SMA^+^ cells (> 60%) within the UUO kidney were from GFP^+^ bone marrow macrophages (Figure 1D).

### Cell transfer studies identify bone marrow macrophages as a source of myofibroblasts in renal fibrosis

**Figure 3:**
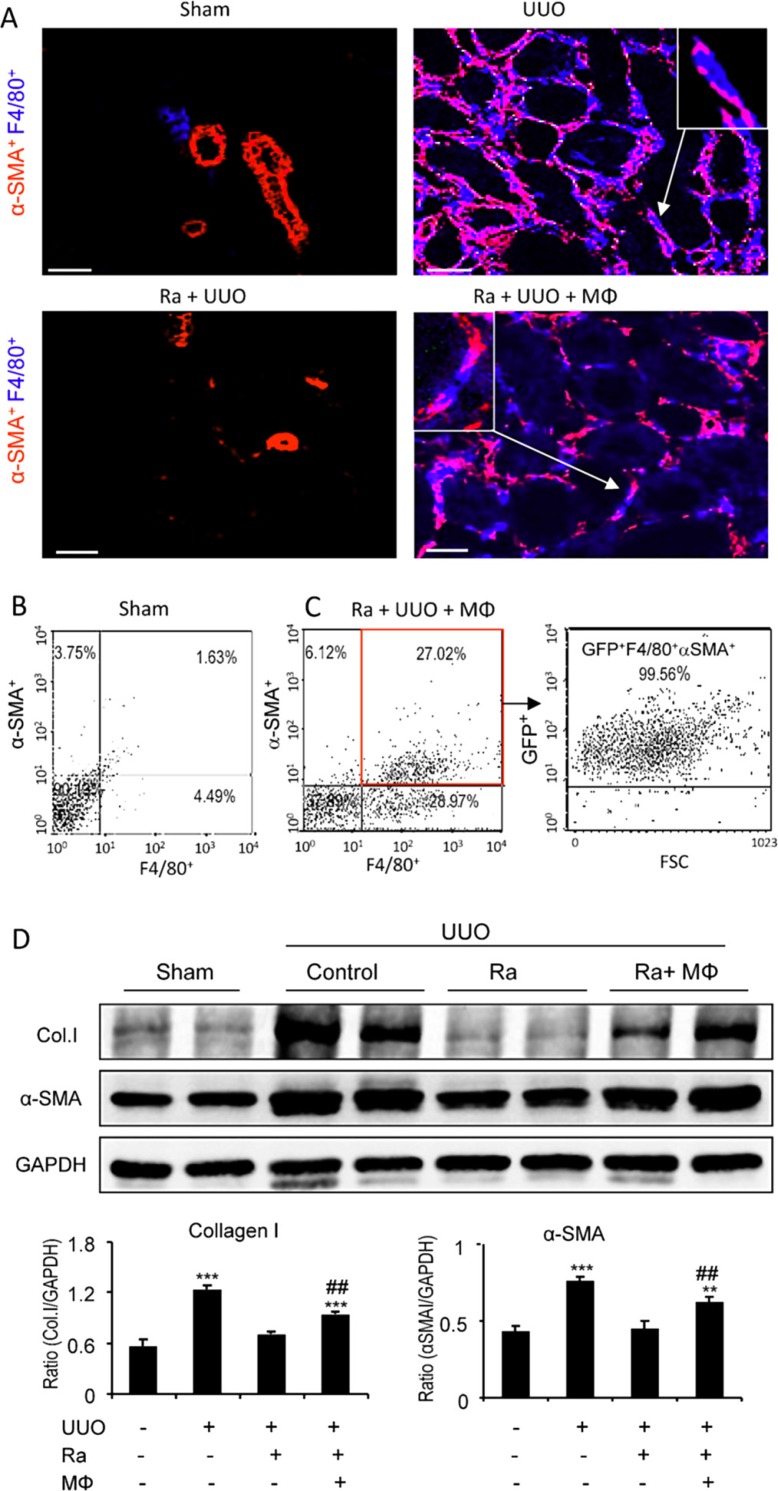
Adoptive transfer identifies bone marrow macrophages as myofibroblast precursors during renal fibrosis Mice underwent lethal irradiation 3 days before UUO surgery, with groups of mice either given no cells (radiation only - Ra) or receiving GFP^+^ bone marrow-derived macrophages (GFP+F4/80+) 1 hr after UUO surgery (radiation plus bone marrow macrophage transfer - BMT) and killed 7 days later. (**A**) Confocal imaging shows that bone marrow depletion by lethal irradiation prevents F4/80^+^ macrophage infiltration and reduces α-SMA^+^ myofibroblast accumulation (Ra+UUO) when compared to the UUO kidney, which is partially restored by GFP^+^ bone marrow macrophage transfer. An example of a GFP^+^α-SMA^+^ cell is shown in the insert. (**B, C**) Flow cytometry shows α-SMA and F4/80 double staining in cells isolated form sham (B) and the UUO kidney following irradiation and transfer of GFP^+^F4/80^+^ bone marrow macrophages (C). Many α-SMA^+^F4/80^+^ cells in the UUO kidney express GFP. (**D**) Western blotting shows increased levels of collagen I and α-SMA proteins in the UUO kidney which is reduced by lethal irradiation but partially restored by transfer of bone marrow macrophages. Graphs show quantification of Western blotting for collage I and α-SMA. Data represent results from groups of 6 animals and each bar resents mean ± SEM. *P < 0.05, compared to sham-operated mice or control cells.***P < 0.001 compared with UUO mice with irradiation. Scale bar, 20 μM.

To formally establish that a subset of myofibroblasts derived directly from bone marrow macrophages, we performed a cell transfer study. Mice underwent lethal irradiation and 3 days later underwent UUO or sham surgery plus adoptive transfer of highly purified GFP^+^ F4/80^+^ bone marrow cells and were killed 7 days later. Irradiation substantially reduced macrophage recruitment and α-SMA^+^ myofibroblast accumulation on day 7 UUO; however, transfer of GFP^+^F4/80^+^ bone marrow cells reconstituted the accumulation of GFP^+^F4/80^+^ macrophages and GFP^+^F4/80^+^α-SMA^+^ myofibroblasts as shown by two-color confocal microscopy and three-color flow cytometric analysis (Figure 3), demonstrating that many of α-SMA^+^ myofibroblasts isolated from the UUO kidney originated from transferred bone marrow macrophages (Figure 3C). Furthermore, the reduction seen in expression of collagen and α-SMA in the UUO kidney due to irradiation was partially reconstituted by the adoptive transfer of GFP^+^F4/80^+^ bone marrow macrophages (Figure 3D).

**Figure 4:**
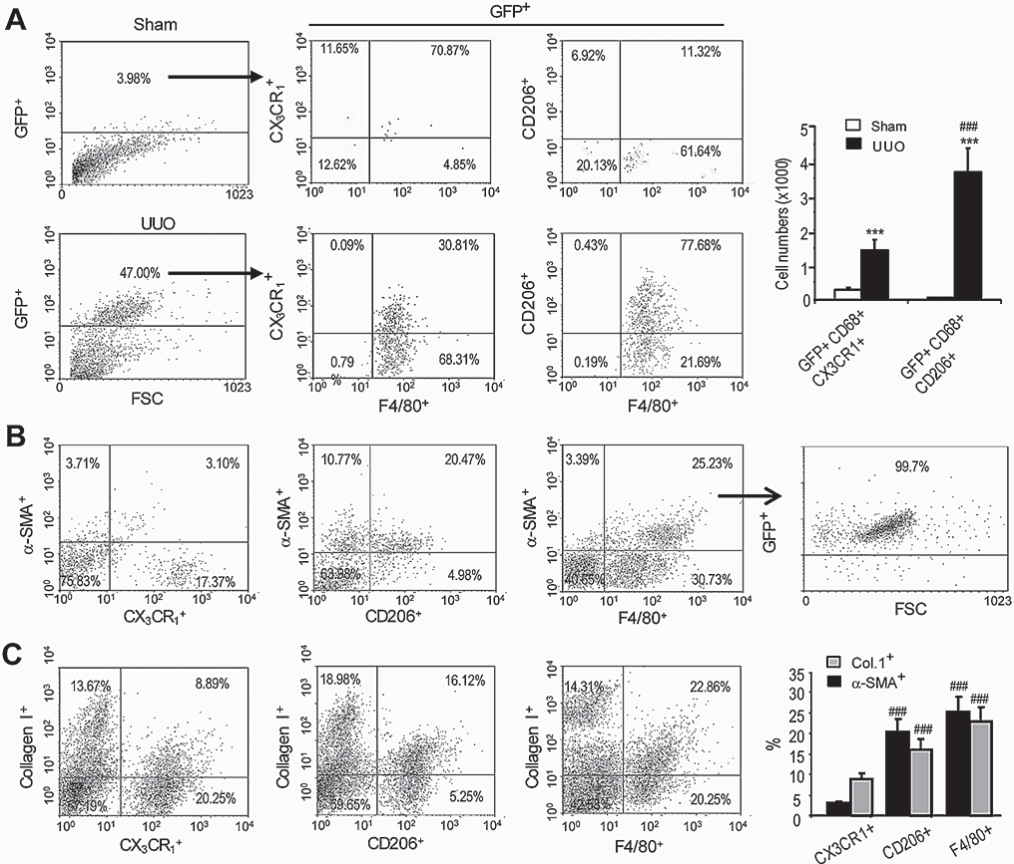
Bone marrow macrophages that transition into myofibroblasts display a predominant M2 phenotype in the UUO kidney Mice underwent lethal irradiation and 3 days later underwent UUO or sham surgery plus adoptive transfer of GFP^+^F4/80^+^ bone marrow cells and were killed 7 days later. (**A**) Flow cytometry of GFP^+^ cells isolated from the day 7 UUO kidney shows that most GFP^+^CD68^+^ cells express the M2 marker, CD206, while a minority express the M1 marker, CX3CR1. In contrast, few GFP^+^ cells were detectable in the sham-operated kidney. (**B**) Analysis of α-SMA^+^ myofibroblasts from the UUO kidney shows 80% of GFP^+^α-SMA^+^F4/80^+^ cells express CD206 while a small population expresses CX3CR1. (**C**) Analysis of collagen I producing cells from the UUO kidney shows more than 65% of collagen I^+^F4/80^+^ cells expressed CD206 while a minority expressed CX3CR1. Data represent results from 5 animals and each bar resents mean ± SEM. ***P < 0.001 as compared to controls; ***P < 0.001 versus CX3CR1+ macrophages.

In further analysis of this study, flow cytometry identified a small number of GFP^+^F4/80^+^ macrophages within the sham-operated kidney and these cells exhibited a predominant M1 phenotype, based on expression of the M1 marker CX3CR1 and lack of expression of the M2 marker CD206 (Figure 4A). In contrast, the majority (> 75%) of GFP^+^F4/80^+^ cells from the UUO kidney expressed CD206 and lacked CX3CR1 (Figure 4A). Importantly, the majority of α-SMA^+^ cells in day 7 UUO kidney that originated from GFP^+^F4/80^+^ bone marrow macrophages had a predominant M2 phenotype (CD206^+^CX3CR1^−^) (Figure 4B). Furthermore, many collagen I producing cells isolated from the UUO kidney were bone marrow-derived GFP^+^F4/80^+^α-SMA^+^ cells with a predominant CD206^+^ M2 phenotype (Figure 4C).

**Figure 5:**
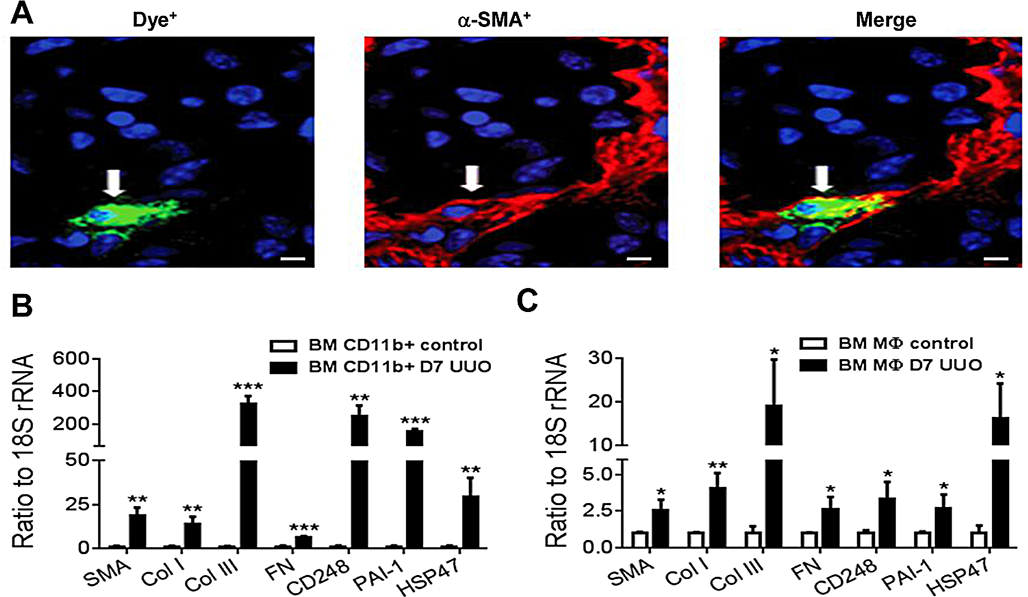
Myofibroblast transition of adoptively transferred bone marrow macrophages in the UUO kidney Dye-labelled CD11b^+^ bone marrow cells were transferred into mice on day 2 following UUO surgery which then were killed on day 7. (**A**) Confocal microscopy shows a dye-labeled macrophage cell (green) in the UUO kidney with co-expression of α-SMA (red). (**B**) Dye-labeled cells were isolated from day 7 UUO kidneys by fluorescence-activated cell sorting and analysed for fibrotic gene expression by real-time PCR in comparison to dye-labelled CD11b^+^ bone marrow cells before transfer (control). (**C**) A similar study using adoptive transfer of dye-labelled cells showed that bone marrow-derived macrophages following a 6 day culture with M-CSF can also home to the UUO kidney and up-regulate the same panel of myofibroblast markers. Data are from 3 groups of mice in which dye-labelled cells were isolated from 2 pooled UUO kidneys. Data are mean ± SD. *P < 0.05, **P < 0.01, ***P < 0.001 compared to control cells. Scale bar, 20 μM.

To further characterize the transition of bone marrow macrophages into myofibroblasts, we performed a second adoptive transfer strategy in which dye-labelled CD11b^+^ bone marrow macrophages were transferred into mice 2 days after UUO surgery. Whilst only small numbers of dye-labelled cells were observed in the day 7 UUO kidney, confocal microscopy identified expression of α-SMA by dye-labelled cells indicating transition towards a myofibroblast phenotype (Figure 5A). Furthermore, PCR analysis of dye-labelled cells isolated from the day 7 UUO kidney demonstrated a clear differentiation towards a myofibroblast phenotype with up-regulation of numerous pro-fibrotic molecules, including α-SMA and collagen I and III compared to the cells initially transferred (Figure 5B). A similar, though less marked, transition response was evident using adoptive transfer of bone marrow-derived macrophages prepared by a standard 6 day culture with M-CSF (Figure 5C).

### Macrophage-myofibroblast transition operates via the TGF-β/Smad3 pathway in renal fibrosis

**Figure 6:**
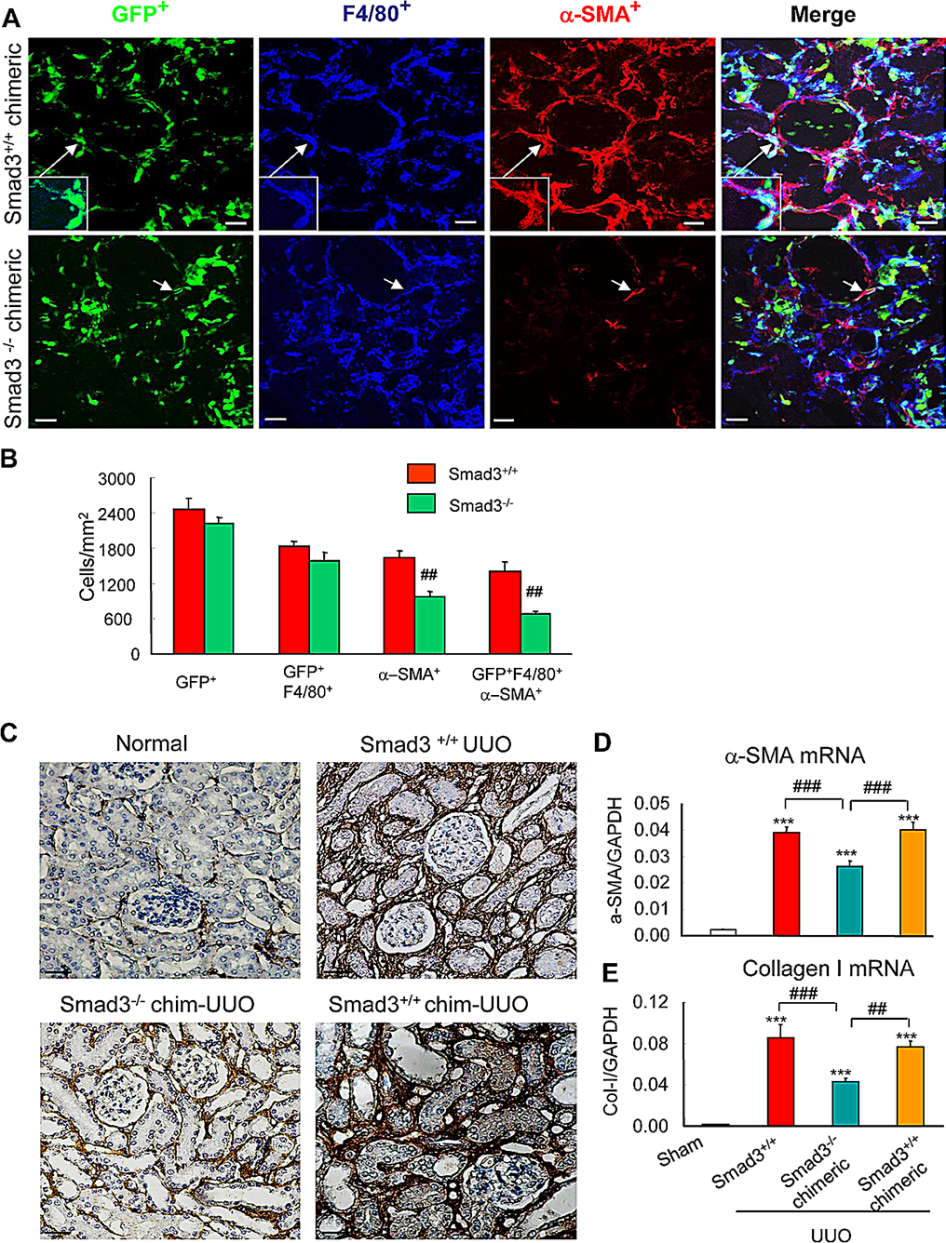
Smad3 is required for macrophage to myofibroblast transition during renal fibrosis (**A**) Wild type mice were lethally irradiated, reconstituted with GFP^+^Smad3^−/−^ or GFP^+^Smad3^+/+^ bone marrow cells and 8 weeks later underwent UUO and were killed 7 days later. Confocal microscopy showing GFP^+^ cells (green), α-SMA^+^ (red, myofibroblasts) and F4/80^+^ (blue, macrophages). GFP^+^Smad3^+/+^ reconstituted mice show numerous infiltrating GFP^+^ cells in the UUO kidney, many of which co-express F4/80 and α-SMA (example in inset). While many GFP^+^ and F4/80^+^ cells are seen in the UUO kidney in mice reconstituted with GFP^+^Smad3^−/−^ bone marrow, there is a marked reduction in GFP^+^α-SMA^+^F4/80^+^ cells. (**B**) Quantification of confocal microscopy analysis in the UUO kidney (*n* = 6). (**C**) Collagen I immunohistochemistry staining of kidney sections. (**D** and **E**) Real time PCR analysis is shown for; (D) α-SMA, and (E) collagen I, mRNA levels. Bars represent mean ± SEM for 6 mice. *P < 0.05, **P < 0.01, ***P < 0.001 versus sham-control; *P < 0.05, **P < 0.01, ***P < 0.001 as indicated. Scale bar, 50 μM.

TGF-β1 can induce transition of epithelial or endothelial cells into myofibroblasts *in vitro* [24–29]; however, whether this operates in the process of macrophage-myofibroblast transition during renal fibrosis is not clear. TGF-β receptors signal via members of the Smad family, of which Smad3 has been most clearly implicated in renal fibrosis [25, 28]. We used chimeric mice to examine TGF-β/Smad3 signalling in the transition of bone-marrow-derived macrophages into myofibroblasts. Irradiated C57/BL6 wild-type mice were reconstituted with either GFP^+^Smad3^−/−^ or GFP^+^Smad3^+/+^ bone marrow cells from C57/BL6 mice for 8 weeks, followed by induction of the UUO model. In the UUO kidney, a majority of α-SMA^+^ myofibroblasts co-expressed GFP and F4/80 in mice reconstituted with GFP^+^Smad3^+/+^ bone marrow (Figure 6A and 6B). By contrast, mice reconstituted with GFP^+^Smad3^−/−^ bone marrow showed a reduction in the overall accumulation of α-SMA^+^ myofibroblasts and a reduction in the proportion of α-SMA^+^ cells co-expressing GFP and F4/80 (Figure 6A and 6B). However, this difference was not due to altered recruitment of GFP^+^F4/80^+^ bone marrow-derived macrophages into the injured kidney which was equivalent in the two groups (Figure 6A and 6B). Thus, the recruited Smad3^− /−^ macrophages failed to transition into myofibroblasts within the fibrosing kidney. This had a functional impact since mice reconstituted with Smad3^−/−^ bone marrow showed a substantial reduction in renal fibrosis on the basis of collagen I deposition in the UUO kidney along with reduced mRNA levels for α-SMA and collagen I (Figure 6C–6E).

### *In vitro* studies of TGF-β1/Smad3 induced macrophage-myofibroblast transition

**Figure 7:**
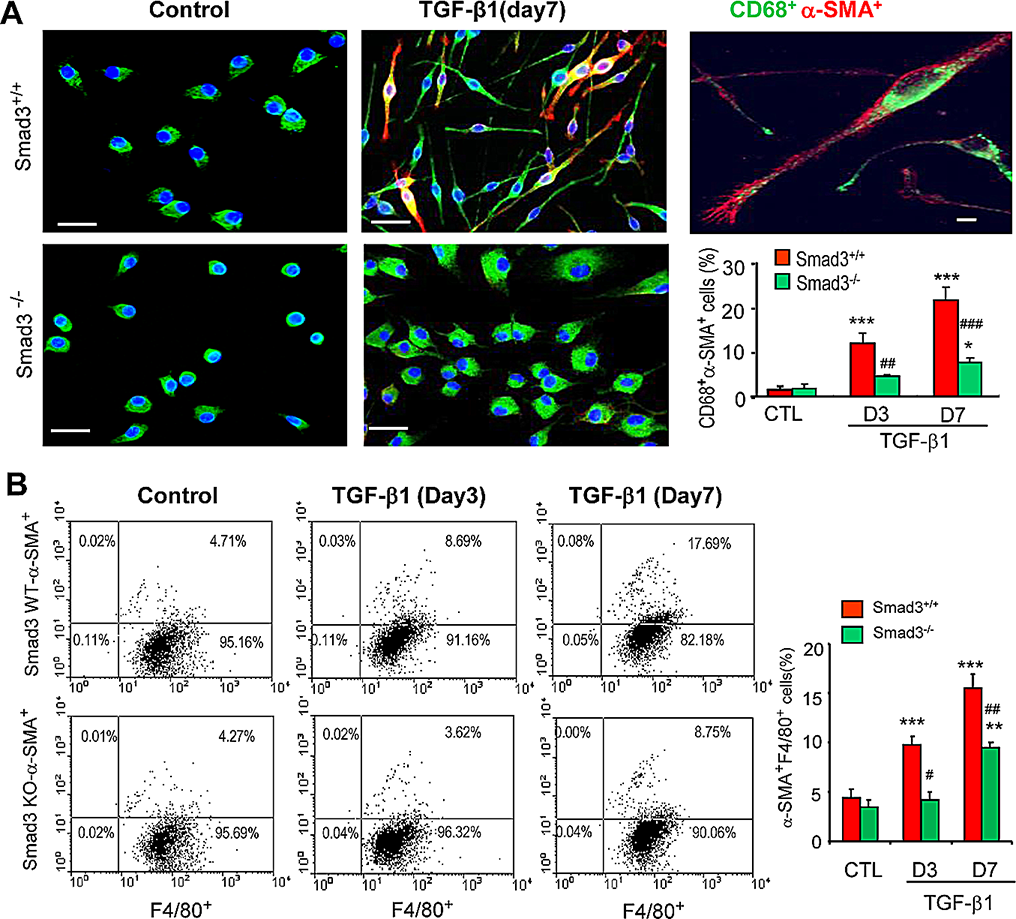
Smad3 is required for macrophage to myofibroblast transition *in vitro* (**A**) F4/80^+^ cells were isolated from the bone marrow of wild type (S3WT) or Smad3^−/−^ (S3KO) mice by fluorescence-activated cell sorting and cultured with M-CSF (50 ng/ml) for 3 or 7 days in the presence or absence of TGF-β1 (5 ng/ml) to induce transition. Immunofluorescence staining for CD68 (green) and α-SMA (red) with nuclear DAPI (blue) counterstain identified CD68^+^α-SMA^+^ cells following TGF-β1 stimulation, which is prominent in S3WT cells but not in S3KO cells. A high power view of a Smad3 WT bone marrow macrophage in transition is shown. A graph shows quantification of the immunofluorescence staining. (**B**) Two-color flow cytometry identified a time-dependent increase in the percentage of F4/80^+^α-SMA^+^ cells following TGF-β1 stimulation in Smad3^+/+^ bone marrow macrophages which is substantially reduced in Smad3^−/−^ macrophages. A graph shows quantification of the flow cytometry analysis. Data represent results from 4 independent *in vitro* experiments and each bar resents mean ± SEM. **P < 0.01, ***P < 0.001 compared to control cells (CTL); *P < 0.05, ***P < 0.01, ***P < 0.001 versus Smad3 WT macrophages. Scale bar, 50 mM.

**Figure 8:**
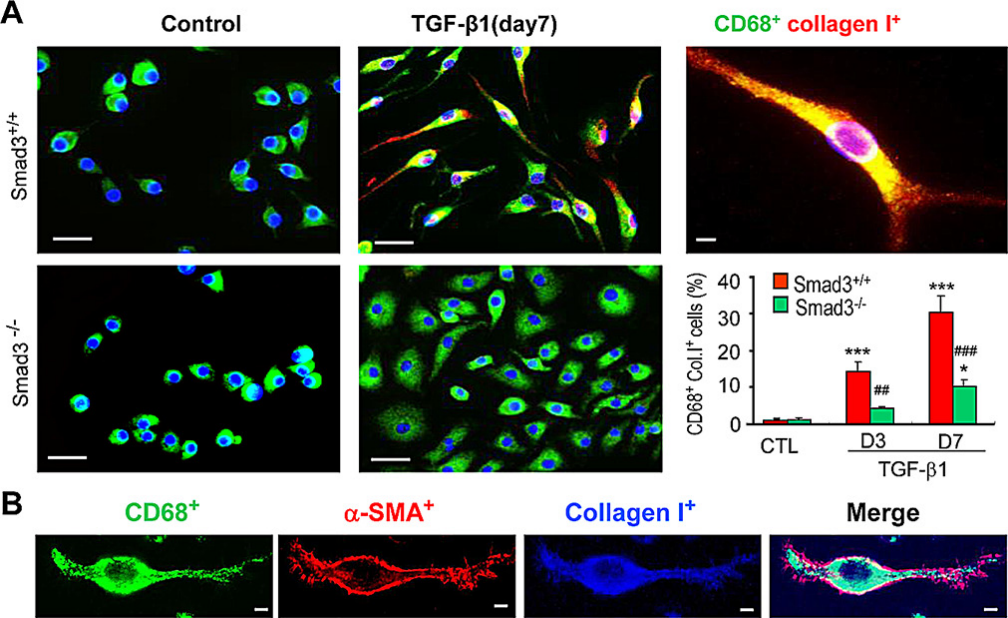
Smad3 is Required for Collagen Production by Transformed Macrophages *in vitro* (**A**) F4/80^+^ cells were isolated from the bone marrow of wild type (S3WT) or Smad3^−/−^ (S3KO) mice and cultured with M-CSF (50ng/ml) for 3 or 7 days in the presence or absence of TGF-β1 (5 ng/ml) to induce transition. Two-colour immunostaining showed collagen I (red) producing macrophages (CD68^+^, green) following TGF-β1 stimulation were much more prominent in Smad3^+/+^ cells compared to Smad3^−/−^ cells. A graph shows quantification of the immunofluorescence staining. (**B**) An example of a collagen-producing macrophage identified by three-color confocal microscopy featuring polarization with *de novo* peripheral α-SMA^+^ actin localization and characteristic head-end, back-end formation. Data represent results from 4 independent *in vitro* experiments and each bar resents mean ± SEM. ***P < 0.001 compared to control cells (CTL); ***P < 0.01, ***P < 0.001 versus Smad3 WT macrophages. Scale bar, 50 μM.

**Figure 9:**
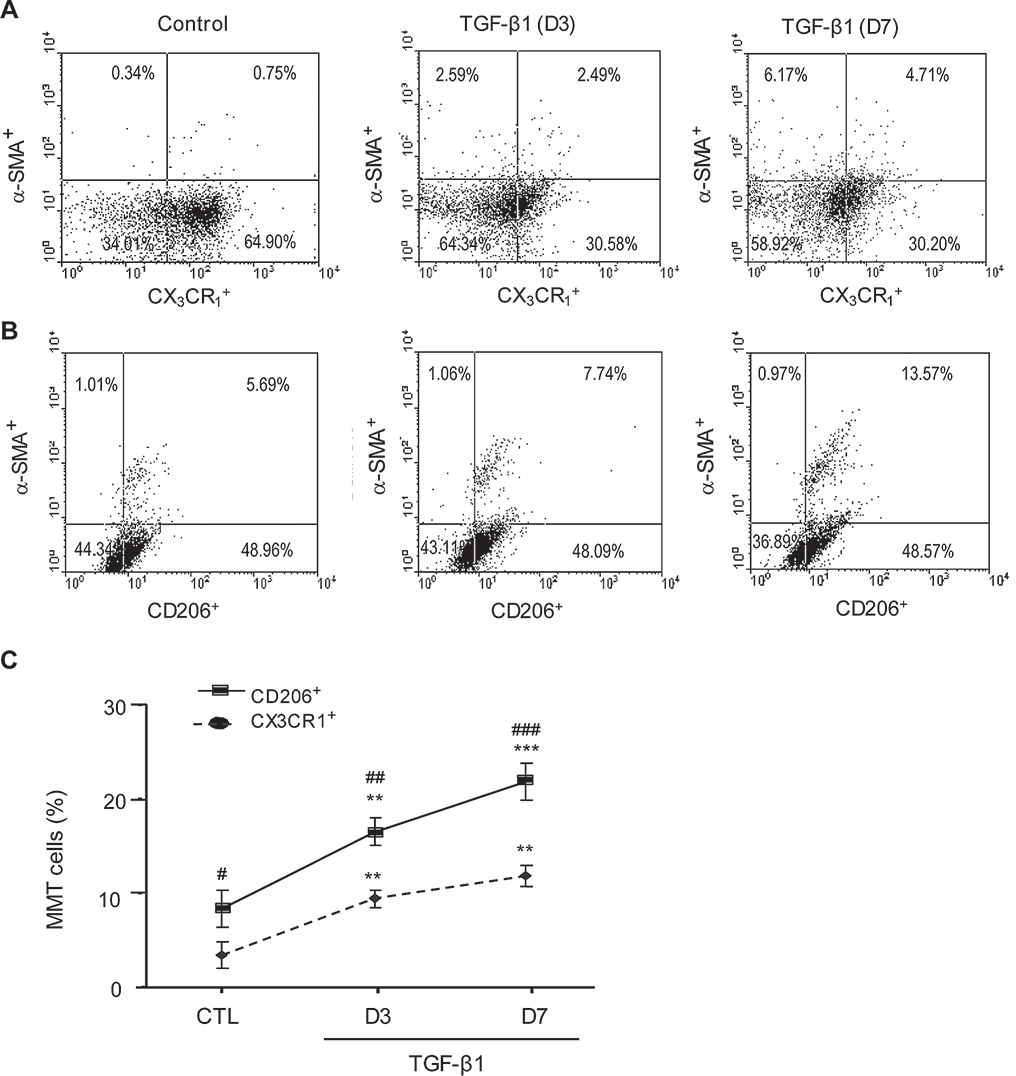
*In vitro* bone marrow macrophages undergoing myofibroblast transition have a predominant M2 phenotype F4/80^+^ cells were isolated from bone marrow and cultured with M-CSF (50 ng/ml) in the presence or absence of TGF-β1 (5 ng/ml) for 3 or 7 days. Flow cytometry identified *de novo* expression of α-SMA in a subset of cultured F4/80^+^ macrophages. The majority of α-SMA^+^ macrophages co-expressed the M2 marker CD206 (> 70%), with a minority expressing the M1 marker, CX3CR1. Data represent results from 5 animals or 4 independent *in vitro* experiments and each bar resents mean ± SEM. **P < 0.01, ***P < 0.001 as compared to control cells (CTL); **P < 0.01,***P < 0.001 versus CX3CR1+ macrophages.

A more detailed analysis of TGF-β1/Smad3 signaling in macrophage to myofibroblast transition was performed using F4/80^+^ cells purified from the bone marrow of Smad3^+/+^ or Smad3^−/−^ mice by fluorescence-activated cell sorting. Culture of bone marrow Smad3^+/+^F4/80^+^ cells for 7 days with M-CSF did not up-regulate expression of α-SMA or collagen I (Figures 7A and 8A). However, the addition of TGF-β1 for 3 or 7 days induced transition of macrophages into collagen-producing myofibroblasts as shown by *de novo* expression of α-SMA (CD68^+^α-SMA^+^) and collagen I (CD68^+^Col I^+^) in Smad3^+/+^ bone marrow macrophages (Figures 7 and 8). Three-color confocal imaging also identified CD68^+^ macrophages expressing α-SMA and collagen I with a characteristic myofibroblast morphology (Figures 7A and 8). In contrast, bone marrow macrophages lacking Smad3 showed substantial resistance to TGF-β1-induced α-SMA and collagen I expression (Figures 7 and 8). Consistent with the *in vivo* studies, most bone marrow macrophages induced to transition into α-SMA^+^ myofibroblasts *in vitro* expressed a predominant CD206+ M2 phenotype (Figure 9).

## DISCUSSION

The identification of bone marrow-derived cells in the obstructed kidney which express α-SMA and collagen I confirms the findings of a number of recent studies. [14–18, 20] We have substantially extended these studies by identifying the macrophage compartment as the source of these bone marrow-derived myofibroblasts. Importantly, we have also demonstrated that MMT is a major source of myofibroblast origin (> 60%) that occurs locally within the fibrosing kidney and is regulated by TGF-β/Smad3 signalling.

There are many distinct cell populations within the bone marrow, three of which have been implicated in modifying renal fibrosis. Bone marrow-derived mesenchymal stem cells exert anti-inflammatory and anti-fibrotic actions in different models of kidney disease. [30, 31]. In contrast, bone marrow-derived fibrocytes and macrophages have been proposed as fibroblast precursor cells in renal fibrosis. [19, 20, 22, 32, 33]. Fibrocytes are a poorly defined off-shoot of the monocyte lineage in which CD45^+^CD34^+^CD11b^+^ monocyte precursors differentiate into a population of collagen I expressing cells which exist in the circulation or within the spleen. [33–35] However, establishing the specific role of fibrocytes in renal fibrosis, or indeed in any type of fibrotic disease, is problematic given the current lack of tools available for lineage tracing of fibrocytes and the major overlap of the commonly used fibrocyte markers (CD45+collagen I+ cells) with other leukocyte populations.

In contrast to previous studies of bone marrow-derived fibroblasts, the current study utilised adoptive transfer strategies to identify the macrophage compartment as the primary source of fibroblast precursors within the bone marrow. The transfer of GFP^+^F4/80^+^ bone marrow macrophages in the UUO model performed in irradiated mice identified these cells as the direct precursor of a subset of myofibroblasts within the obstructed kidney. In the second adoptive transfer model, analysis of dye-labelled CD11b^+^ bone marrow macrophages before and after entry into the obstructed kidney indicates that these cells underwent transition into collagen-producing myofibroblasts within the fibrosing kidney. This argues against circulating or splenic fibrocytes as the main source of bone marrow-derived fibroblasts [15, 33]. The consistent findings across the bone marrow chimera and two independent transfer studies provide a strong argument for bone marrow macrophages as the main precursor for bone marrow-derived fibroblasts in interstitial renal fibrosis. These findings are supported by a recent study in which macrophages isolated from kidneys undergoing fibrosis secondary to glycerol-induced acute kidney injury were shown to express pro-fibrotic transcripts, including collagen III and fibronectin [32]. Finally, these *in vivo* findings are supported by *in vitro* experiments establishing that TGF-β1 is capable of inducing collagen-producing MMT cells (F4/80+ α-SMA+ collagen I+) in bone marrow-derived macrophages, although macrophages may also participate in the collagen internalization and degradation in the disease status.

An interesting finding was that most macrophages undergoing MMT in the fibrosing kidney expressed the M2 marker, CD206, with only a minor subset expressing the M1 marker. Analysis of the MMT response using cultured bone marrow macrophages also showed a greater response of macrophages expressing M2 compared to M1 markers. It is well known that M1- type inflammatory macrophages in acute inflammatory lesions can differentiate into reparative or pro-fibrotic M2-type macrophages in active fibrotic lesions [36–39]. However, mechanisms regulating the distinct functions of M2 macrophages during tissue repair or fibrosis remain largely unclear. Transfer of splenic M2 macrophages protects against, but transfer of bone marrow–derived M2 macrophages promotes renal fibrosis, [40] suggesting that M2 macrophages from bone marrow have a greater capacity to undergo MMT compared to M1 macrophages, whereas, M2 macrophages from spleen are reparative. The distinct role of bone marrow versus spleen–derived M2 macrophages in renal fibrosis may be associated with the local proliferating activities of macrophages. Indeed, recent studies have shown that bone marrow-derived M2-type pro-fibrotic macrophages are highly proliferative, which may contribute to promote renal fibrosis in the UUO kidney and the adriamycin nephropathy [40–42]. Interestingly, systemic depletion of monocytes and macrophages using liposomal clodronate, but not diphtheria toxin, attenuates acute kidney injury, suggesting a distinct role between the circulating and resident macrophages during acute or repairing process [43]. Although results from the present study could not clearly distinguish the fibrotic response caused by circulating or resident macrophages, the finding of more than 90% of MMT cells co-expressing GFP in chimeric mice demonstrated a bone marrow origin and may play a direct and indirect role during renal fibrosis. [44]

It is well accepted that TGF-β1 is a key growth factor which drives tissue fibrosis. Canonical TGF-β1 signalling operates via TGF-β receptors and Smad2/3/4 transcription factors [25]. The protection of Smad3 gene knockout mice in models of tissue fibrosis indicates that TGF-β/Smad3 signalling is pro-fibrotic, while conditional Smad2 deficiency promotes fibrosis, indicating opposing effects of Smad2 and Smad3 [25, 28, 45, 46]. The present study identifies that Smad3 was required for the efficient transition of recruited macrophages into collagen I-producing α-SMA+ myofibroblasts within the injured kidney. In addition, the protection seen in Smad3^−/−^ chimeric mice provides evidence that bone marrow-derived macrophages make a substantial contribution to the development of renal fibrosis via the MMT process that is regulated by TGF-β/Smad3 signaling. Thus, the present study provides a mechanistic link between MMT as a source of myofibroblasts and the central role for the TGF-β/Smad3 signalling pathway in tissue fibrosis. However, this finding does not exclude a significant contribution of pericytes, fibrocytes, EMT or EndoMT as other sources of myofibroblasts during tissue fibrosis. It is also possible that other fibrogenic pathways may also contribute in part to this MMT process during renal fibrosis.

**Figure 10:**
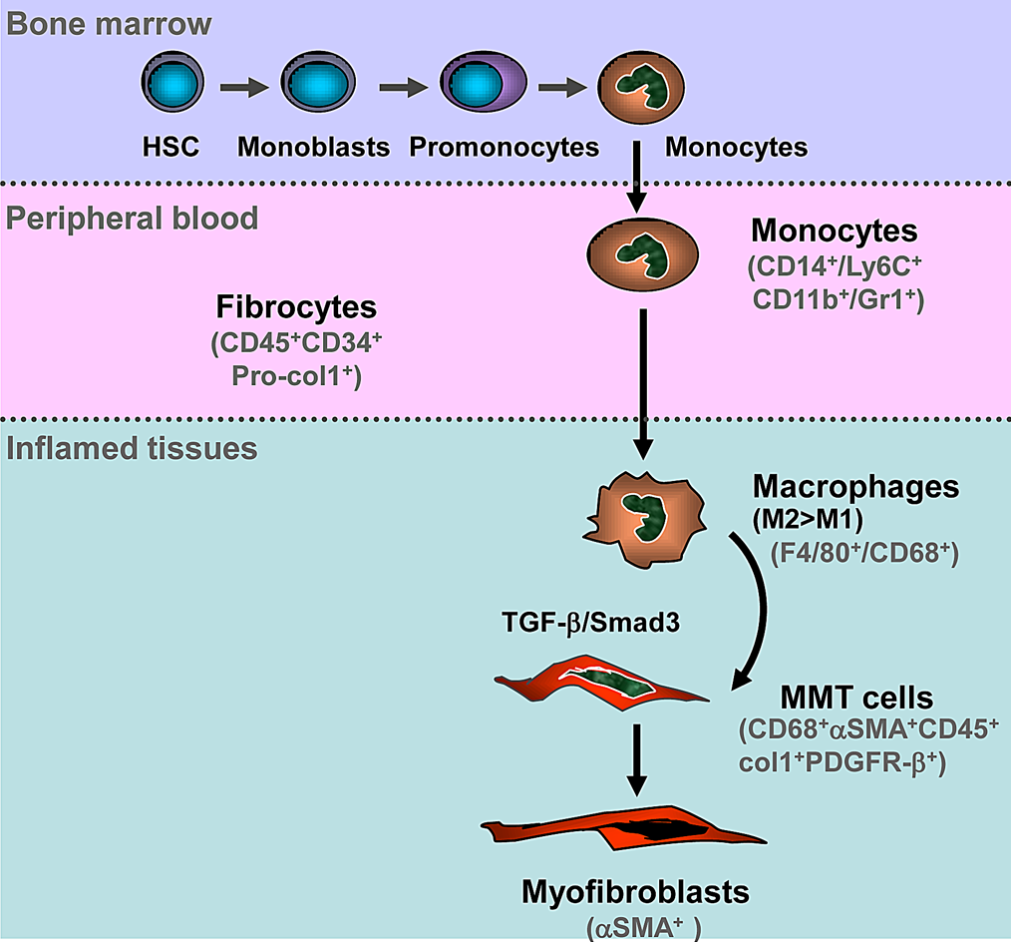
Schematic Diagram of Macrophage-Myofibroblast Transition (MMT) in Tissue Fibrosis Hematopoietic stem cells (HSC) can differentiate into monocytes in the bone marrow. Blood monocytes entering the injured tissue can differentiate into an M2 pro-fibrotic phenotype, either directly or via an M1 pro-inflammatory phenotype. TGF-β/Smad3 signalling then drives macrophage transition into collagen-producing α-SMA^+^ myofibroblasts via the process of MMT.

In conclusion, as shown in Figure 10, we identify bone marrow-derived macrophages, via the process of MMT, as an important source of collagen producing α-SMA^+^ myofibroblasts which accumulate in active fibrotic lesions in experimental kidney disease. This process operates via the TGF-β/Smad3 signalling pathway. These findings suggest that targeting the MMT pathway may represent as a novel therapeutic target for the treatment of chronic diseases associated with progressive fibrosis.

## MATERIALS AND METHODS

### Animals and experimental models

GFP^+^ mice (C57BL/6) were crossed with Smad3^+/−^ mice (C57BL/6) to generate GFP^+^Smad3^+/+^ or GFP^+^Smad3^−/−^ mice. Chimeric mice were generated by lethally irradiation of C57BL/6 mice followed by transfusion of 5 × 10^6^ GFP^+^Smad3^−/−^ or GFP^+^Smad3^+/+^ bone marrow cells and waiting 8 weeks to develop full (> 95%) bone marrow chimerism.^47^ UUO surgery was performed by ligation of the left ureter and mice killed 7 days later. [10, 28] Groups of 6 mice of both genders, aged 8–10 weeks, were studied. Experiments were approved by the Animal Experimentation Ethics Committee of The Chinese University of Hong Kong.

### Macrophage transfer studies

In the first set of studies, F4/80^+^ macrophages were purified (> 99% purity) by fluorescence-activated cell sorting (FACS) from GFP^+^ C57BL6 bone marrow cells cultured for 7 days in DMEM/F12 media supplied with 10% FBS and 50 ng/ml M-CSF for 7 days. C57BL/6 mice were lethally irradiated, UUO surgery performed 3 days later and 1 hr after surgery mice were given 1 × 10^6^ GFP^+^F4/80^+^ cells by intravenous injection 1 hr after surgery. Mice were killed on day 7.

In the second set of studies, CD11b^+^ cells were isolated from fresh bone marrow by FACS, labelled *in vitro* with SP-DiOC_18_ (Sigma-Aldrich, Castle Hill, NSW, Australia) and 5 × 10^6^ cells were injected intravenously per mouse (*n* = 6) on day 2 after UUO surgery. Mice were killed on day 7 after UUO and the dye-labelled cells examined by confocal microscopy or isolated from 2 pooled UUO kidneys following enzyme digestion using FACS for subsequent RNA extraction and PCR analysis. In addition, macrophages were prepared by culturing bone marrow cells for 6 days with M-CSF, dye-labelled and injected into mice on day 2 after UUO surgery and then killed on day 7 and analyzed as above. These studies were performed at Monash Medical Centre, Clayton, Australia with the approval of the local animal ethics committee.

### Immunofluorescence and confocal microscopy

Immunofluorescence and confocal microscopy were performed on frozen sections using: rat anti-mouse F4/80 (Serotec Ltd, Oxford, UK); Cy3-labeled mouse anti-mouse α-SMA (Sigma, St. Louis, MO); goat anti-collagen I (Southern Tech, Birmingham, AL, USA); FITC-labeled rat anti-mouse CD206 (Serotec Ltd, Oxford, UK); rabbit anti-CX3CR1 (Prosci incorporated, Poway, CA); Cy5-labeled goat anti-rabbit antibody (Invitrogen, Carlsbad, CA, USA) and Alexa555-labeled donkey anti-goat antibody (Invitrogen, Carlsbad, CA). Sections were examined using a fluorescent microscope (Model: Axioplan2 imaging, Carl Zeiss, Oberkoche, Germany) or confocal microscope (Model: LSM 510 META, Carl Zeiss). In addition, a single MMT cell co-expressing F4/80 and α-SMA antigens was examined by a Z-stack image. Single, double, or triple positive cells were counted in 10 high-power fields (× 40) per section by means of a 0.0625 mm^2^ graticule fitted in the eyepiece of the microscope and expressed as cells per mm^2^.

### Flow cytometry

Single cells were isolated from both normal and diseased kidneys using enzyme-digestion and analyzed by flow cytometry as previously described.^47^ After permeabilization, cells were incubated with FITC-conjugated rat anti-mouse F4/80 (eBioscience) or rat anti-mouse CD11b (Serotec) followed by Cy5-conjugated goat anti-rat IgG (Millpore), PE-conjugated anti-α-SMA (R & D, Minneapolis, MN, USA), FITC-labeled rat anti-mouse CD206 (Serotec) or rabbit anti-CX3CR1(Prosci Inc). Collagen I was stained with rabbit anti-mouse collagen I (Millpore) followed by the Cy5 labeled goat anti-rabbit Ig (Invitrogen). Cells incubated with isotype-matched irrelevant control antibodies and unstained cells were used as negative controls. Cells were detected by a FACS Calibur flow cytometer (BD Biosciences) and analyzed using Cellquest software.

### Culture of bone marrow-derived macrophages

Bone marrow cells were isolated from Smad3^+/+^ or Smad3^−/−^ mice by flushing the femur and tibia with DMEM/F12 medium, dispersing the cells and isolating F4/80^+^ cells via FACS (> 99% purity). Bone marrow F4/80^+^ cells were cultured for 3 or 7 days with DMEM/F12 containing 10%FBS and 50 ng/ml M-CSF in the presence or absence of TGF-β1 (5 ng/ml). Cells then were analyzed by confocal microscopy or flow cytometry for expression of F4/80, α-SMA, collagen I, and M1 (CX3CR1) or M2 (CD206) markers. Cells were cultured in Smooth Muscle Differentiation medium (Invitrogen) with M-CSF in the presence or absence of TGF-β1 (5 ng/ml) for 3 or 7 days.

### Histology and immunohistochemistry

Immunostaining was performed on 3 μm paraffin sections using a microwave-based antigen retrieval technique [26, 27, 48].

### Real-time PCR and Western blot analysis

Total RNA was isolated from kidney tissue or isolated cells using the RNeasy Isolation Kit (Qiagen, Valencia, CA). Real time RT-PCR was performed as previously described [26, 27, 49, 50]. Western Blotting of kidney tissue lysates was performed as previously described [26, 27, 50].

### Statistical analysis

Data are expressed as the mean ± SEM and analyzed using one-way analysis of variance (ANOVA), followed by Tukey's post-hoc test using GraphPad Prism 5.

## SUPPLEMENTARY VIDEO



## References

[1] Meng XM, Nikolic-Paterson DJ, Lan HY (2014). Inflammatory processes in renal fibrosis. Nat Rev Nephrol.

[2] Hewitson TD (2009). Renal tubulointerstitial fibrosis: common but never simple. Am J Physiol Renal Physiol.

[3] Klingberg F, Hinz B, White ES (2013). The myofibroblast matrix: implications for tissue repair and fibrosis. J Pathol.

[4] Mack M, Yanagita M (2015). Origin of myofibroblasts and cellular events triggering fibrosis. Kidney Int.

[5] Jinde K, Nikolic-Paterson DJ, Huang XR, Sakai H, Kurokawa K, Atkins RC, Lan HY (2001). Tubular phenotypic change in progressive tubulointerstitial fibrosis in human glomerulonephritis. Am J Kid Dis.

[6] Ng YY, Huang TP, Yang WC, Chen ZP, Yang AH, Mu W, Nikolic-Paterson DJ, Atkins RC, Lan HY (1998). Tubular epithelial-myofibroblast transdifferentiation in progressive tubulointerstitial fibrosis in 5/6 nephrectomized rats. Kidney Int.

[7] Iwano M, Plieth D, Danoff TM, Xue C, Okada H, Neilson EG (2002). Evidence that fibroblasts derive from epithelium during tissue fibrosis. J Clin Invest.

[8] Liu Y (2010). New insights into epithelial-mesenchymal transition in kidney fibrosis. J Am Soc Nephrol.

[9] Zeisberg EM, Potenta SE, Sugimoto H, Zeisberg M, Kalluri R (2008). Fibroblasts in kidney fibrosis emerge via endothelial-to-mesenchymal transition. J Am Soc Nephrol.

[10] Zeisberg EM, Tarnavski O, Zeisberg M, Dorfman AL, McMullen JR, Gustafsson E, Chandraker A, Yuan X, Pu WT, Roberts AB, Neilson EG, Sayegh MH, Izumo S (2007). Endothelial-to-mesenchymal transition contributes to cardiac fibrosis. Nat Med.

[11] Li J, Qu X, Bertram JF (2009). Endothelial-myofibroblast transition contributes to the early development of diabetic renal interstitial fibrosis in streptozotocin-induced diabetic mice. Am J Pathol.

[12] Grgic I, Duffield JS, Humphreys BD (2012). The origin of interstitial myofibroblasts in chronic kidney disease. Pediatr Nephrol.

[13] Humphreys BD, Lin SL, Kobayashi A, Hudson TE, Nowlin BT, Bonventre JV, Valerius MT, McMahon AP, Duffield JS (2010). Fate tracing reveals the pericyte and not epithelial origin of myofibroblasts in kidney fibrosis. Am J Pathol.

[14] Li J, Deane JA, Campanale NV, Bertram JF, Ricardo SD (2007). The contribution of bone marrow-derived cells to the development of renal interstitial fibrosis. Stem Cells.

[15] Jang HS, Kim JI, Han SJ, Park KM (2014). The recruitment and subsequent proliferation of bone marrow-derived cells in postischemic kidney are important to the progression of fibrosis. Am J Physiol Renal Physiol.

[16] Broekema M, Harmsen MC, van Luyn MJ, Koerts JA, Petersen AH, van Kooten TG, van Goor H, Navis G, Popa ER (2007). Bone marrow-derived myofibroblasts contribute to the renal interstitial myofibroblast population and produce procollagen I after ischemia/reperfusion in rats. J Am Soc Nephrol.

[17] LeBleu VS TG, O'Connell J, Teng Y, Cooke VG, Woda C, Sugimoto H, Kalluri R (2013). Origin and function of myofibroblasts in kidney fibrosis. Nat Med.

[18] Jang HS, Kim JI, Jung KJ, Kim J, Han KH, Park KM (2013). Bone marrow-derived cells play a major role in kidney fibrosis via proliferation and differentiation in the infiltrated site. Biochimica Biophysica Acta.

[19] Phua YL, Martel N, Pennisi DJ, Little MH, Wilkinson L (2013). Distinct sites of renal fibrosis in Crim1 mutant mice arise from multiple cellular origins. J Pathol.

[20] Yang J, Lin SC, Chen G, He L, Hu Z, Chan L, Trial J, Entman ML, Wang Y (2013). Adiponectin promotes monocyte-to-fibroblast transition in renal fibrosis. J Am Soc Nephrol.

[21] Lin SL, Kisseleva T, Brenner DA, Duffield JS (2008). Pericytes and perivascular fibroblasts are the primary source of collagen-producing cells in obstructive fibrosis of the kidney. Am J Pathol.

[22] Chen G, Lin SC, Chen J, He L, Dong F, Xu J, Han S, Du J, Entman ML, Wang Y (2011). CXCL16 recruits bone marrow-derived fibroblast precursors in renal fibrosis. J Am Soc Nephrol.

[23] Xia Y, Yan J, Jin X, Entman ML, Wang Y (2014). The chemokine receptor CXCR6 contributes to recruitment of bone marrow-derived fibroblast precursors in renal fibrosis. Kidney Int.

[24] Fan JM, Ng YY, Hill PA, Nikolic-Paterson DJ, Mu W, Atkins RC, Lan HY (1999). Transforming growth factor-beta regulates tubular epithelial-myofibroblast transdifferentiation *in vitro*. Kidney Int.

[25] Meng XM, Chung AC, Lan HY (2013). Role of the TGF-beta/BMP-7/Smad pathways in renal diseases. Clin Sci.

[26] Huang XR, Chung AC, Yang F, Yue W, Deng C, Lau CP, Tse HF, Lan HY (2010). Smad3 mediates cardiac inflammation and fibrosis in angiotensin II-induced hypertensive cardiac remodeling. Hypertension.

[27] Yang F, Huang XR, Chung AC, Hou CC, Lai KN, Lan HY (2010). Essential role for Smad3 in angiotensin II-induced tubular epithelial-mesenchymal transition. J Pathol.

[28] Sato M, Muragaki Y, Saika S, Roberts AB, Ooshima A (2003). Targeted disruption of TGF-beta1/Smad3 signaling protects against renal tubulointerstitial fibrosis induced by unilateral ureteral obstruction. J Clin Invest.

[29] Li J, Qu X, Yao J, Caruana G, Ricardo SD, Yamamoto Y, Yamamoto H, Bertram JF (2010). Blockade of endothelial-mesenchymal transition by a Smad3 inhibitor delays the early development of streptozotocin-induced diabetic nephropathy. Diabetes.

[30] Asanuma H, Vanderbrink BA, Campbell MT, Hile KL, Zhang H, Meldrum DR, Meldrum KK (2011). Arterially delivered mesenchymal stem cells prevent obstruction-induced renal fibrosis. J Surg Res.

[31] Wu HJ, Yiu WH, Li RX, Wong DW, Leung JC, Chan LY, Zhang Y, Lian Q, Lin M, Tse HF, Lai KN, Tang SC (2014). Mesenchymal stem cells modulate albumin-induced renal tubular inflammation and fibrosis. PloS One.

[32] Belliere J, Casemayou A, Ducasse L, Zakaroff-Girard A, Martins F, Iacovoni JS, Guilbeau-Frugier C, Buffin-Meyer B, Pipy B, Chauveau D, Schanstra JP, Bascands JL (2015). Specific macrophage subtypes influence the progression of rhabdomyolysis-induced kidney injury. J Am Soc Nephrol.

[33] Reich B, Schmidbauer K, Rodriguez Gomez M, Johannes Hermann F, Gobel N, Bruhl H, Ketelsen I, Talke Y, Mack M (2013). Fibrocytes develop outside the kidney but contribute to renal fibrosis in a mouse model. Kidney Int.

[34] Bucala R, Spiegel LA, Chesney J, Hogan M, Cerami A (1994). Circulating fibrocytes define a new leukocyte subpopulation that mediates tissue repair. Mol Med.

[35] Strieter RM, Keeley EC, Hughes MA, Burdick MD, Mehrad B (2009). The role of circulating mesenchymal progenitor cells (fibrocytes) in the pathogenesis of pulmonary fibrosis. J Leuk Biol.

[36] Ikezumi Y, Suzuki T, Karasawa T, Hasegawa H, Yamada T, Imai N, Narita I, Kawachi H, Polkinghorne KR, Nikolic-Paterson DJ, Uchiyama M (2011). Identification of alternatively activated macrophages in new-onset paediatric and adult immunoglobulin A nephropathy: potential role in mesangial matrix expansion. Histopathology.

[37] Ikezumi Y, Suzuki T, Yamada T, Hasegawa H, Kaneko U, Hara M, Yanagihara T, Nikolic-Paterson DJ, Saitoh A (2015). Alternatively activated macrophages in the pathogenesis of chronic kidney allograft injury. Pediatr Nephrol.

[38] Lawrence T, Natoli G (2011). Transcriptional regulation of macrophage polarization: enabling diversity with identity. Nat Rev Immunol.

[39] Murray PJ, Wynn TA (2011). Protective and pathogenic functions of macrophage subsets. Nature Rev Immunol.

[40] Cao Q, Wang Y, Zheng D, Sun Y, Wang C, Wang XM, Lee VW, Wang Y, Zheng G, Tan TK, Wang YM, Alexander SI, Harris DC (2014). Failed renoprotection by alternatively activated bone marrow macrophages is due to a proliferation-dependent phenotype switch *in vivo*. Kidney Int.

[41] Engel DR, Krause TA, Snelgrove SL, Thiebes S, Hickey MJ, Boor P, Kitching AR, Kurts C (2015). CX3CR1 reduces kidney fibrosis by inhibiting local proliferation of profibrotic macrophages. J Immunol.

[42] Pan B, Liu G, Jiang Z, Zheng D (2015). Regulation of renal fibrosis by macrophage polarization. Cell Physiol Biochem.

[43] Ferenbach DA, Sheldrake TA, Dhaliwal K, Kipari TM, Marson LP, Kluth DC, Hughes J (2012). Macrophage/monocyte depletion by clodronate, but not diphtheria toxin, improves renal ischemia/reperfusion injury in mice. Kidney Int.

[44] Meng XM, Nikolic-Paterson DJ, Lan HY (2014). Inflammatory processes in renal fibrosis. Nat Rev Nephrol.

[45] Wang W, Huang XR, Canlas E, Oka K, Truong LD, Deng C, Bhowmick NA, Ju W, Bottinger EP, Lan HY (2006). Essential role of Smad3 in angiotensin II-induced vascular fibrosis. Circulation Res.

[46] Meng XM, Huang XR, Chung AC, Qin W, Shao X, Igarashi P, Ju W, Bottinger EP, Lan HY (2010). Smad2 protects against TGF-beta/Smad3-mediated renal fibrosis. J Am Soc Nephrol.

[47] Lin SL, Castano AP, Nowlin BT, Lupher ML, Duffield JS (2009). Bone marrow Ly6Chigh monocytes are selectively recruited to injured kidney and differentiate into functionally distinct populations. J Immunol.

[48] Lan HY, Mu W, Nikolic-Paterson DJ, Atkins RC (1995). A novel, simple, reliable, and sensitive method for multiple immunoenzyme staining: use of microwave oven heating to block antibody crossreactivity and retrieve antigens. J Histochem Cytochem.

[49] Ma FY, Tesch GH, Ozols E, Xie M, Schneider MD, Nikolic-Paterson DJ (2011). TGF-beta1-activated kinase-1 regulates inflammation and fibrosis in the obstructed kidney. Am J Physiol Renal Physiol.

[50] Xiao J, Meng XM, Huang XR, Chung AC, Feng YL, Hui DS, Yu CM, Sung JJ, Lan HY (2012). miR-29 inhibits bleomycin-induced pulmonary fibrosis in mice. Mol Ther.

